# Heat shock protein amplification improves cerebellar myelination in the *Npc1*^nih^ mouse model

**DOI:** 10.1016/j.ebiom.2022.104374

**Published:** 2022-11-28

**Authors:** James Gray, María E. Fernández-Suárez, Maysa Falah, David Smith, Claire Smith, Ecem Kaya, Ashley M. Palmer, Cathrine K. Fog, Thomas Kirkegaard, Frances M. Platt

**Affiliations:** a Department of Pharmacology, University of Oxford, Oxford OX1 3QT, UK; b Orphazyme A/S, Ole Maaloes Vej 3, Copenhagen DK-2200, Denmark

**Keywords:** lysosomal storage diseases, Niemann-Pick disease type C, NPC, neurodegeneration, myelination, heat shock proteins, HSP70, cholesterol, glycosphingolipids, Fyn kinase, arimoclomol, bimoclomol, BALB/cNctr-*Npc1*^*m1N*^/J, *Npc1*^*nih*^, mice

## Abstract

**Background:**

Niemann-Pick disease type C (NPC) is a rare prematurely fatal lysosomal lipid storage disease with limited therapeutic options. The prominent neuropathological hallmarks include hypomyelination and cerebellar atrophy. We previously demonstrated the efficacy of recombinant human heat shock protein 70 (rhHSP70) in preclinical models of the disease. It reduced glycosphingolipid levels in the central nervous system (CNS), improving cerebellar myelination and improved behavioural phenotypes in *Npc1*^nih^ (*Npc1*^*−/−*^) mice. Furthermore, treatment with arimoclomol, a well-characterised HSP amplifier, attenuated lysosomal storage in NPC patient fibroblasts and improved neurological symptoms in *Npc1*^*−/−*^ mice. Taken together, these findings prompted the investigation of the effects of HSP amplification on CNS myelination.

**Methods:**

We administered bimoclomol daily or rhHSP70 6 times per week to *Npc1*^*−/−*^ (BALB/cNctr-*Npc1*^*m1N*^/J, also named *Npc1*^nih^) mice by intraperitoneal injection from P7 through P34 to investigate the impact on CNS myelination. The Src-kinase inhibitor saracatinib was administered with/without bimoclomol twice daily to explore the contribution of Fyn kinase to bimoclomol's effects.

**Findings:**

Treatment with either bimoclomol or rhHSP70 improved myelination and increased the numbers of mature oligodendrocytes (OLs) as well as the ratio of active-to-inactive forms of phosphorylated Fyn kinase in the cerebellum of *Npc1*^*−/−*^ mice. Additionally, treatment with bimoclomol preserved cerebellar weight, an effect that was abrogated when co-administered with saracatinib, an inhibitor of Fyn kinase. Bimoclomol-treated mice also exhibited increased numbers of immature OLs within the cortex.

**Interpretation:**

These data increase our understanding of the mechanisms by which HSP70 regulates myelination and provide further support for the clinical development of HSP-amplifying therapies in the treatment of NPC.

**Funding:**

Funding for this study was provided by Orphazyme A/S (Copenhagen, Denmark) and a Pathfinder Award from The Wellcome Trust.

**Research in context**

**Evidence before the study**

Niemann-Pick disease type C (NPC) is a progressive neurodegenerative lysosomal storage disease caused by autosomal recessive mutations in either the *NPC1* or *NPC2* genes. The current therapeutic options for treating the neuropathology and associated clinical manifestations of NPC are extremely limited. We have previously shown that treatment with recombinant human heat shock protein 70 (rhHSP70) in *Npc1*^nih^ (*Npc1*^*−/−*^) mice reduced central nervous system (CNS) glycosphingolipid (GSL) levels and improved myelination and behavioural phenotypes related to cerebellar atrophy. Treatment with arimoclomol, an HSP amplifier, additionally decreased lysosomal storage in NPC patient fibroblasts and neurological symptoms in *Npc1*^*−/−*^ mice, thereby prompting us to further investigate the roles of HSP amplification in ameliorating the defective myelination observed within the CNS in NPC.

**Added value of the study**

Bimoclomol is an analogue of the well-characterised HSP amplifier, arimoclomol, an orally available, CNS-penetrant, small molecule currently in late-stage clinical development for the treatment of NPC. A recently completed clinical phase II/III trial with arimoclomol in NPC reported positive results showing a marked reduction in disease progression and significant effect on biomarkers of target engagement, specifically HSP70. The program has subsequently received Fast Track and Breakthrough Therapy designations with the US Food and Drug Administration. The data herein suggest that treatment with HSP-amplifying compounds may be a promising therapeutic strategy for improving white matter thickness and counteracting hypomyelination, which is associated with the clinical manifestations of ataxia and impaired ocular-motor functioning in NPC, and therefore provide further mechanistic detail on the disease-modifying effects of HSP amplification by this class of compounds.

**Implications of all the available evidence**

In summary, the available evidence suggests that HSP-amplifying compounds may be an effective strategy for treating the neuropathological lesions and subsequent clinical manifestations of NPC.

## Introduction

Niemann-Pick disease type C (NPC) is a neurovisceral lysosomal lipid storage disease caused by autosomal recessive mutations in either the *NPC1* (95% of patients) or *NPC2* genes leading to deficiency of NPC1 or NPC2.^1^, ^2^, ^3^ NPC is a lipid trafficking disorder, characterised by the accumulation of unesterified cholesterol, glycosphingolipids (GSLs), sphingosine and sphingomyelin, within the late endosome/lysosome.^3^^,^^4^ NPC disease caused by mutations in *NPC1* or *NPC2* is clinically similar and patients display a broad range of symptoms with varying ages of onset and severity of disease.^1^^,^^5^ The majority (90%) of patients exhibit progressive neurodegenerative disease,^3^^,^^6^ of which the prominent neuropathological hallmarks include myelination defects and neuronal loss. Patients display hypomyelination of white matter^7^, ^8^, ^9^ as well as atrophy of the cerebellum^8^, ^9^, ^10^ and forebrain,^8^^,^^11^^,^^12^ which have been associated with the clinical manifestations of disease including ataxia^9^ and impairments in saccadic gain.^9^^,^^11^

The *Npc1*^nih^ (hereafter referred to as *Npc1*^*−/−*^) mouse arose from a spontaneous insertional mutation resulting in undetectable levels of NPC1 protein^13^ and recapitulates many of the neuropathological hallmarks observed in infantile onset NPC, including hypomyelination^14^, ^15^, ^16^, ^17^ and cerebellar degeneration.^18^^,^^19^ Within the CNS, myelination is achieved by oligodendrocytes (OLs), a type of glial cell that forms myelin from specialised membrane processes, ensheathing multiple axons to produce a compact multi-lamellar sheath and allowing the proper function and maintenance of neurons.^20^^,^^21^ During development, the OL progenitor cells proliferate and migrate throughout the CNS prior to differentiating into mature, myelin forming OLs.^20^^,^^22^ Myelin is a highly specialised extension of the plasma membrane, rich in lipid species such as cholesterol, sphingomyelin, and GSLs as well as myelin-specific glycoproteins, including myelin basic protein (MBP).^20^

The initiation and extension of myelin is a tightly regulated process involving the interaction between axonal ligands, laminin-2 and L1 cell adhesion molecule (L1), and OL membrane receptors, α6β1-integrin with dystroglycan and contactin, respectively.^23^^,^^24^ The OL membrane receptors mediate control over the proto-oncogene tyrosine-protein kinase Fyn (Fyn), a critical regulator of myelination.^24^, ^25^, ^26^ Fyn is a member of the v-src sarcoma (Schmidt–Ruppin A-2) viral oncogene homolog (Src) family of non-receptor tyrosine kinases, and the activity of Fyn kinase is primarily controlled through phosphorylation of two tyrosine residues, tyrosine 418 (Y418) and tyrosine 531 (Y531).^24^^,^^26^ The Y418 residue is activatory and stimulates the kinase activity of Fyn.^27^ The phosphorylation of Y418 is controlled through the interaction between contactin and L1.^26^ Fyn kinase Y531 residue is inhibitory and, when phosphorylated, prevents substrate binding within the Src Homology 2 (SH2) domain.^27^^,^^28^ The dephosphorylation of this residue is required for full function of Fyn and is coordinated by binding of α6β1-integrin with dystroglycan and laminin-2.^26^^,^^29^ Fyn directly drives expression of MBP,^30^ differentiation of OLs,^31^ and the initiation and extension of myelin sheaths.^25^ Loss of *Npc1* in neurons leads to stalled OL maturation and failure of myelination, an observation that was associated with decreased activation of Fyn kinase (Y418 low:Y531 high).^32^ Furthermore, conditional loss of *Npc1* in OLs resulted in delayed myelination at early stages of development whereas aged mice with conditional loss of *Npc1* in OLs exhibited a breakdown in myelin and subsequent degeneration of cerebellar Purkinje neurons,^32^ suggesting that *Npc1* is required for both the formation and maintenance of CNS myelin. *Npc1*^*−/−*^ mice display a markedly similar hypomyelination phenotype to both the Fyn kinase knockout mouse model^25^ and a dystrophic (dy) mouse model (*dy/dy*) in which expression of the laminin 2 subunit is severely reduced.^33^

There is no cure for NPC and the current treatment options are extremely limited.^34^^,^^35^ The only approved therapy for the treatment of NPC in Europe and other countries outside the US is miglustat, a GSL biosynthesis inhibitor, which inhibits glucosylceramide synthase.^2^^,^^36^ In *Npc1*^*−/−*^ mice, treatment with miglustat led to reductions in CNS GSL levels and improved Purkinje cell survival, behavioural phenotypes and lifespan.^37^ Furthermore, treatment with miglustat slowed the loss of white matter in adolescent and adult-onset NPC patients^38^^,^^39^ and has also been shown to stabilise disease progression and extend lifespan.^40^^,^^41^ The heat shock protein 70 (HSP70) is capable of correcting lysosomal accumulation of sphingomyelin in Niemann-Pick disease type A and B (acid sphingomyelinase deficiency) fibroblasts through an intra-lysosomal mechanism of enhancing the activity of mutated acid sphingomyelinase.^42^ We recently demonstrated that recombinant human HSP70 (rhHSP70) also affects other sphingolipid hydrolases, suggesting a broader effect on lysosomal lipid metabolism.^43^ Additionally, treatment with rhHSP70 in *Npc1*^*−/−*^ mice reduced GSL accumulation within the CNS and improved the behavioural phenotypes associated with NPC. A prominent histopathological observation in the brains of *Npc1*^*−/−*^ mice treated with rhHSP70 was a clear improvement in cerebellar myelination.^43^ Furthermore, the small molecule arimoclomol, an orally available, CNS-penetrant, HSP amplifier, was also able to significantly improve the neurological symptoms and extend the lifespan in *Npc1*^*−/−*^ mice.^43^ A recently completed clinical phase II/III trial with arimoclomol for the treatment of NPC reported positive results showing a reduction in disease progression, as measured by the primary endpoint, the 5-domain NPC Clinical Severity Scale (Clinicaltrials.gov identifier: NCT02612129).^44^ In addition, the biomarker data showed a clear biological effect of arimoclomol in terms of target engagement and lipid biomarkers, as evidenced by an increase in the levels of circulating HSP70 and reductions in the levels of accumulated unesterified cholesterol in skin and blood cells in patients treated with arimoclomol.^44^

In the current study, we investigated the impact of heat shock protein amplification on CNS myelination in the *Npc1*^*−/−*^ mouse model by treating the mice with the arimoclomol analogue, bimoclomol. Similar to arimoclomol, bimoclomol has been shown to sustain HSF1 activation and amplify HSP70 levels.^45^, ^46^, ^47^, ^48^ We hypothesised that treatment with bimoclomol would lead to improvements in myelin formation. To test this hypothesis, *Npc1*^*−/−*^ mice were treated with bimoclomol or rhHSP70 from P7 to P34. Treatment with either bimoclomol or rhHSP70 improved cerebellar myelination in *Npc1*^*−/−*^ mice. This observation was accompanied by a normalisation in the numbers of mature OLs as well as increases in the ratio of active-to-inactive forms of phosphorylated Fyn kinase in the cerebellum, both of which were reduced in vehicle-treated *Npc1*^*−/−*^ mice. Additionally, *Npc1*^*−/−*^ mice treated with bimoclomol exhibited improvements in cerebellar weights, but not when treatment was administered in combination with saracatinib, a Src family kinase inhibitor capable of inhibiting Fyn kinase activity.^49^ Finally, treatment with bimoclomol also increased the numbers of immature OLs within the cortex. Taken together, these findings suggest that amplification of HSPs may improve the hypomyelination observed in *Npc1*^*−/−*^ mice by increasing the numbers of mature OLs required for myelination, potentially via increased Fyn kinase-mediated signalling to the OLs and/or an expansion of the immature OL population.

## Methods

### Ethics

All experiments were conducted using protocols approved by the UK Home Office Animal Scientific Procedures Act, 1986. All animal usage was in compliance with the ARRIVE guidelines.

### Animal studies

BALB/cNctr-*Npc1*^*m1N*^/J mice (termed *Npc1*^*−/−*^ mice, also known as *Npc1*^nih^ mice; RRID: IMSR_JAX:003092)^50^ were generated by heterozygote brother/sister matings obtained from Jackson Laboratory (Charles River, UK) and genotyped as previously described.^43^ Mice were bred and housed in individually ventilated cages (IVCs; Thoren, Hazleton, PA, USA) under non-sterile conditions containing Bcell8 bedding (Anibed, France) and given *ad libitum* access to food (i.e., standard chow) and water. Each IVC housed a total of two litters or up to five adult mice. The animals were maintained on a 12:12 light: dark cycle. Mice were assigned to treatment based on gender and genotyping result without access to information on weight or physical appearance. Individual mice were weighed prior to administration of treatments at postnatal day 7 (P7), and those mice weighing a minimum of 3.5 g were included in the study. Baseline characteristics, including weight and sex, were generally balanced, and the numbers of mice per treatment group were based on previous studies.^43^ *Npc1*^*−/−*^ mice were intraperitoneally (IP) injected 6 times per week in the morning (between 8:00 am and 9:00 am) from P7 to P34 with recombinant human His-tagged HSP70 at 1.5 mg/kg of body weight (*Npc1*^*−/−*^ + rhHSP70; n = 18) or bimoclomol daily at 10 mg/kg of body weight (*Npc1*^*−/−*^ + bimoclomol; n = 17), both from Orphazyme A/S, and dissolved in phosphate buffered saline (PBS). Bimoclomol and arimoclomol are hydroxylamine compounds that amplify heat shock proteins, including HSP70, with the structural difference being the presence of an oxide group on arimoclomol.^47^ The dosing regimen was selected to give a stable exposure to treatment that would be expected to reflect patient dosing, and the weekly dosage was the same as previously used.^43^ The control group was comprised of *Npc1*^*−/−*^ mice IP injected daily with PBS (*Npc1*^*−/−*^ + PBS; n = 18). Untreated *Npc1*^*+/+*^ littermates (n = 16) served as comparators. For the Fyn kinase inhibition experiments, saracatinib^51^ (MedChemExpress) and bimoclomol were IP injected daily at 10 mg/kg of body weight either alone (n = 6) or in combination (n = 6) and dissolved in hydroxypropyl methylcellulose (0.5%, Sigma–Aldrich) and polysorbate 80 (0.1%, Sigma–Aldrich). Controls (n = 6) were injected with vehicle alone. Each treatment group contained separate cohorts of *Npc1*^*+/+*^ and *Npc1*^*−/−*^ mice. Due to the high clearance and low solubility of saracatinib, the daily dosage for all treatments was split with half administered in the morning (between 8:00 am and 9:00 am) and half in the afternoon (between 4:00 pm and 5:00 pm). All IP injections were administered in a sterile hood housed in the procedure room.

Mice were killed at 35 days of age with 800 mg/kg pentobarbital IP. For biochemical analysis, mice were transcardially perfused with ice-cold PBS. The brains were removed and dissected into cerebellum, corpus callosum, or cortex, snap frozen on dry ice, and stored at −80 °C. For immunofluorescence, mice were transcardially perfused with 10 mL PBS followed by 40 mL 4% paraformaldehyde (PFA) in PBS. Brains were removed and post-fixed overnight in 4% PFA before washing three times in PBS and cryoprotecting in 30% sucrose solution. For electron microscopy, mice were transcardially perfused with 10 mL PBS followed by 100 mL of fixative solution containing 4% PFA, 2.5% glutaraldehyde, and 1% picric acid. Brains were removed and washed overnight in 0.1 M phosphate buffer with gentle agitation.

### Immunofluorescence

Brains were cut into 6 series of floating sagittal sections at 20 μm on a Bright OTF cryostat. Sections were blocked in PBS containing 0.3% triton-X with 2% normal goat serum before overnight staining with primary antibodies against MBP (mouse monoclonal IgG1; BioLegend Cat# 836504, RRID: AB_2616694), oligodendrocyte marker O4 (mouse monoclonal IgM; Millipore Cat# MAB345, RRID: AB_11213138), and the pi form of glutathione s-transferase (pi-GST; rabbit polyclonal; Enzo Life Sciences Cat# ADI-MSA-102-E, RRID: AB_2039146) at 1:200, 1:400, and 1:500, respectively, in the same blocking solution at 4 °C. After washing in PBS, conjugated secondary antibodies raised in goat against mouse IgG1 (Alexa Fluor 488; Thermo Fisher Scientific Cat# A-21121, RRID: AB_2535764), mouse IgM (Alexa Fluor 568; Thermo Fisher Scientific Cat# A-21043, RRID: AB_2535712), and rabbit IgG (Alexa Fluor 568; Thermo Fisher Scientific Cat# A-11011, RRID: AB_143157) were each applied at 1:1000 for 2 h at room temperature. Following three washes in PBS, sections were then counterstained with DAPI (300 nM in PBS, Sigma–Aldrich) for 30 min before washing three times with PBS. Sections were mounted onto Superfrost™ slides, air-dried overnight, and protected from light before addition of coverslips with ProLong® Gold mounting medium. Confocal images were acquired using a Leica-SP8 confocal microscope with an LD 20× water objective.

### Electron microscopy

Coronal sections of cerebellum were cut at 70 μm thickness using a VT1000s vibratome, (Leica Instruments) into 6 serial sections and washed three times in 0.1 M phosphate buffer. Sections were incubated with 1% osmium tetroxide for 1 h before dehydrating in a series of washes from 50% to 100% ethanol. Sections were then incubated with a 1% uranyl acetate solution for 40 min before further dehydration in a series of washes from 50% to 100% ethanol. Sections were impregnated with Durcupan resin (Sigma–Aldrich) before baking overnight at 60 °C. For each condition, the cerebellar lobule VIII was dissected from the tissue and re-embedded in resin blocks. Ultrathin sections (70 nm) were cut using an Ultracut UCT ultramicrotome (Leica Instruments) and mounted onto grids. Prior to electron microscopy, grids were post-stained with lead citrate for 3 min at room temperature before air-drying. Imaging was done using a Tecnai 12 (FEI) transmission electron microscopy (TEM) microscope operated at 120 kV and equipped with a Oneview camera (Gatan).

### Western blotting

For protein expression, dissected tissues were weighed prior to homogenisation in 40 volumes of RIPA buffer containing 1× Halt™ protease inhibitor cocktail (Thermo Fisher) and phosphatase inhibitor cocktail (Invitrogen). Homogenates were agitated on ice for 20 min before centrifugation at 12,000*g* for 10 min. The protein concentration of lysates was determined using the bicinchoninic acid (BCA) assay (Sigma–Aldrich). For Western blotting, samples each containing 15 μg protein were separated using Nu-PAGE 4–12% bis-tris gradient gels (BioRad) before transferring onto PVDF membranes (Biorad) and blocking in PBS containing 0.5% Tween-20 and 5% de-fatted milk powder for 1 h at room temperature. Blots were probed with primary mouse monoclonal antibodies against MBP (BioLegend Cat# 836504, RRID: AB_2616694) or myelin 2′,3′-cyclic nucleotide-3′-phosphodiesterase (CNPase; BioLegend Cat# 836404, RRID: AB_2566639) at 1:2500 or 1:500, respectively, overnight at 4 °C in blocking solution and washed three times before probing with horseradish peroxidase (HRP) conjugated secondary antibodies raised in sheep or donkey against mouse (GE Healthcare Cat# NXA931, RRID: AB_772209) or rabbit (GE Healthcare Cat# NA934, RRID: AB_772206) at 1:25,000. Protein loading was quantified using a mouse monoclonal anti-β actin HRP conjugated antibody at 1:25,000 (Sigma–Aldrich Cat# A3854, RRID: AB_262011). For saracatinib experiments, due to the effects of saracatinib on actin-ring formation,^52^ blots were normalised by Coomassie staining as previously described.^53^ Briefly, after processing, blots were washed three times with PBS containing 0.5% Tween-20 and incubated for 1 min in 1:1 methanol:water containing 0.1% Coomassie before transferring to 5:4:1 methanol:water:acetic acid for 20 min. Blots were then rinsed with water and allowed to air-dry. Immunoreactivity was visualised using the Pierce ECL western blotting substrate (Thermo Fisher). Total protein, loading controls and protein expression were quantified using ImageLab software (version 3.0, BioRad).

### Immunoprecipitation

Protein A Dynabeads (Invitrogen) were incubated with a rabbit polyclonal antibody against Fyn (Santa Cruz Biotechnology Cat# sc-16, RRID: AB_631528) at 1:2000 for 10 min at room temperature. Unbound antibody was washed from beads with PBS and the beads were conjugated using BS3 (Thermo Scientific) according to manufacturer's instructions before resuspending in original bead volume in PBS containing 0.1% Tween-20. For immunoprecipitation of Fyn, cerebellar homogenates were adjusted to 1 mg/mL protein in RIPA buffer containing Halt protease and phosphatase inhibitors. Protein (200 μg) was then incubated with 40 μL of the Dynabead-antibody complex at 4 °C for 1 h. Following incubation, the unbound fraction was collected, and the beads washed 3 times before incubating at 70 °C for 10 min in Laemmli buffer without β-mercaptoethanol. The supernatant was separated from the beads and boiled for 5 min in the presence of β-mercaptoethanol. Bound and unbound fractions were then separated by electrophoresis as described above. Blotted membranes were blocked in PBS with 0.5% Tween-20 containing 5% BSA for 1 h at room temperature. Blots were then probed with primary antibodies against phosphorylated Src (Y418) (rabbit polyclonal; Millipore Cat# 07-909, RRID: AB_568805), Fyn (phospho Y530) and Yes (phospho Y537) (rabbit monoclonal; Abcam, Cat# ab188319), and total Fyn (mouse monoclonal; Abcam Cat# ab1881, RRID: AB_2232153) at 1:1000, 1:5000, and 1:1000 overnight, respectively, before probing with secondary antibodies as described previously. Blots were developed with Pierce SuperSignal West-Femto substrate (Thermo Fisher).

### Image analysis

All image analysis, counts, and quantifications were performed using ImageJ (FIJI) software (version 2.0.0, ImageJ). For quantification of MBP staining, the exterior of the MBP-positive white matter region was outlined, and the total area calculated as a proportion of the total lobular area (as assessed by DAPI staining). Counts of pi-GST- and O4-positive cells were performed manually and adjusted for the total area per field of view of deep cerebellar white matter, corpus callosum, or cortex as applicable. The quantification of proportions of myelinated fibres was performed from 6,800× magnified TEM images of cerebellar white matter (minimum of 500 axons/animal). Axons were identified as single membrane ensheathed structures containing multiple bands of neurofilament, either with or without myelin ensheathment. Measurements of the g-ratio were taken from randomly selected myelinated axons (minimum 50/animal) of TEM images at 12,000× magnification. Axonal and myelin diameter was estimated by dividing the total circumference by pi. The g-ratio was calculated by dividing the estimated axonal diameter by the estimated axonal diameter plus myelin diameter.

### Statistical analysis

Data were compiled in Microsoft Excel for Mac (version 15.37, Microsoft). Statistical analyses were performed using Prism (version 7.0, GraphPad) on log-transformed values to better satisfy the model assumptions for analysis of variance (ANOVA); a full description of the methods is detailed in each of the figure legends. The comparator group was not included in the statistical analyses, except where indicated, as we have previously shown that *Npc1*^*−/−*^ mice exhibit hypomyelination and significantly reduced levels of MBP compared with *Npc1*^*+/+*^ mice.^43^ Therefore, we used the *a priori* comparisons between vehicle (PBS)-treated *Npc1*^*−/−*^ mice and *Npc1*^*−/−*^ mice treated with either rhHSP70 or bimoclomol to assess the effects of treatments. Multiplicity was adjusted using Dunnett's method (∗*p* < 0.05, ∗∗*p* < 0.01, ∗∗∗*p* < 0.001, ∗∗∗∗*p* < 0.0001).

## Results

### Amplification of heat shock proteins increases myelin basic protein levels *in vivo*

**Fig. 1:**
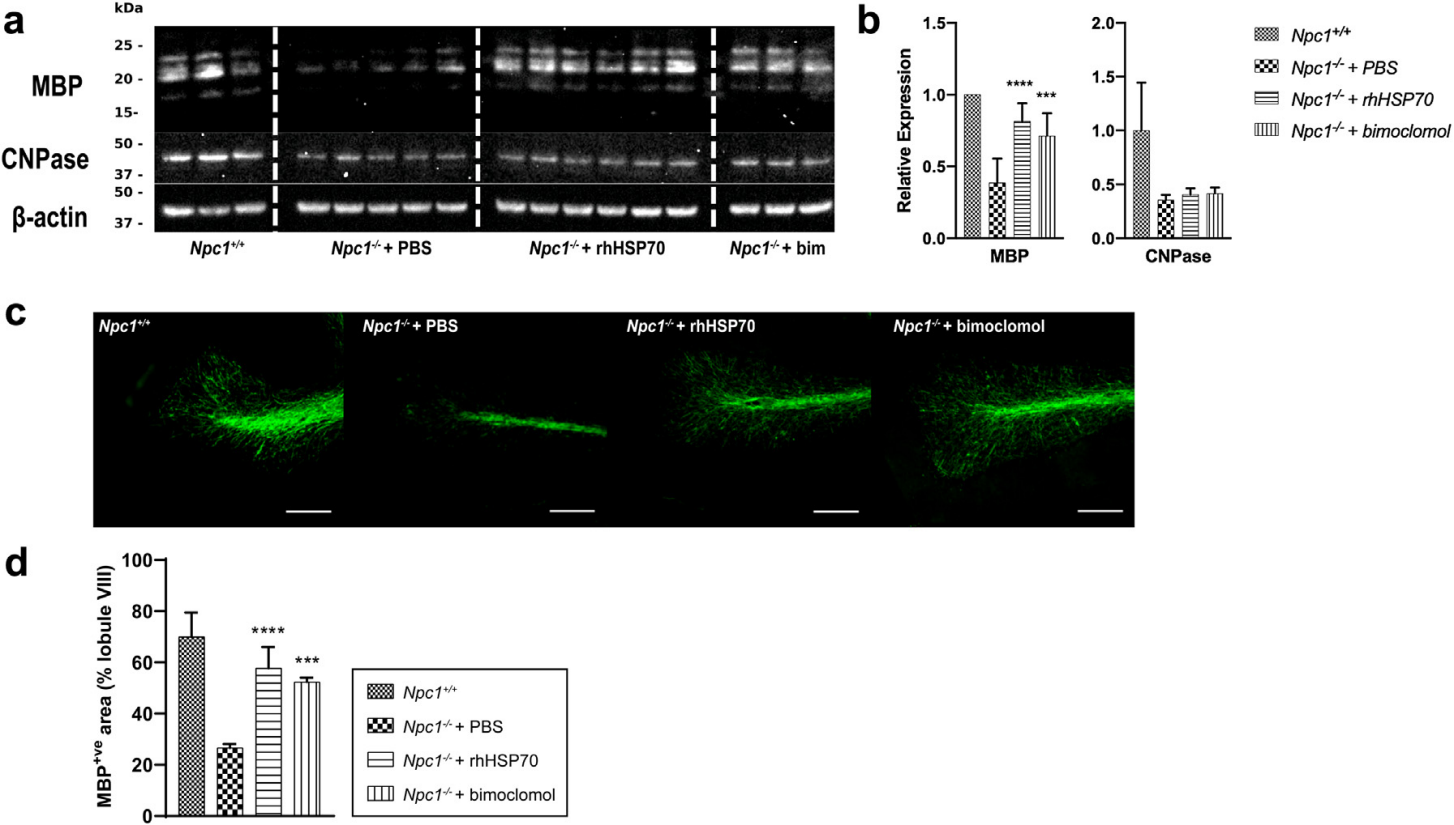
**Amplification of heat shock proteins increases myelin basic protein levels *in vivo***. Western blot analysis of MBP and CNPase levels in the a) cerebellum of *Npc1*^*−/−*^ mice at P35 following treatment with PBS (vehicle), rhHSP70, or bimoclomol (bim). Untreated *Npc1*^*+/+*^ mice were included as comparators. β-actin served as a loading control. The bar graph in b) shows cerebellar levels of MBP & CNPase in treated *Npc1*^*−/−*^ mice relative to those levels in comparators. Number of mice per group (MBP expression): *Npc1*^*+/+*^, n = 4; *Npc1*^*−/−*^ + PBS, n = 6; *Npc1*^*−/−*^ + rhHSP70, n = 6; *Npc1*^*−/−*^ + bimoclomol (Bim), n = 3. In both the comparator (*Npc1*^*+/+*^ mice) and control (*Npc1*^*−/−*^ mice treated with PBS) groups, two mice were assayed less than three times (one mouse was assayed twice and another was assayed once). Data are presented as the mean relative expression values of the mice in each treatment group + standard deviation (SD) of three separate blots. CNPase expression: *Npc1*^*+/+*^, n = 3; *Npc1*^*−/−*^ + PBS, n = 5; *Npc1*^*−/−*^ + rhHSP70, n = 6; *Npc1*^*−/−*^ + bimoclomol, n = 3. c) Representative images of cerebellar lobule VIII stained for MBP in *Npc1*^*+/+*^ mice and *Npc1*^*−/−*^ mice treated with PBS, rhHSP70, or bimoclomol at P35. Scale bar represents 100 μm. d) MBP-positive area served as a proxy for white matter area and was quantified by normalising the MBP-stained area in c) to the total lobular area as assessed by DAPI staining. Data are shown as mean + SD of MBP-positive area from three images per animal from a total of n = 2 animals per treatment group. For the analyses shown in b) and d), effects of treatments were analysed by a nested one-way ANOVA on log-transformed values and multiplicity was adjusted using Dunnett's method (n = 2 comparisons; ∗*p* < 0.05, ∗∗*p* < 0.01, ∗∗∗*p* < 0.001, ∗∗∗∗*p* < 0.0001).

We previously showed that treatment with rhHSP70 significantly increased MBP levels in the cerebellum of *Npc1*^*−/−*^ mice when administered from three weeks of age.^43^ As myelination begins before P7 in the murine CNS and is significantly advanced by 14 days postnatally,^15^^,^^17^ we treated *Npc1*^*−/−*^ mice with 1.5 mg/kg rhHSP70, six times per week, or 10 mg/kg bimoclomol, daily, from P7 until P34. This treatment window corresponds to the period of myelin formation in the human CNS, which begins shortly after birth, peaks at around one year of age, and, in some cortical regions, continues into early adulthood.^20^^,^^54^ The expression of myelin-specific proteins, CNPase and MBP, in the cerebellum of five-week-old mice (P35) was measured by Western blotting. Treatment with either rhHSP70 or bimoclomol significantly increased MBP expression in the cerebellum of *Npc1*^*−/−*^ mice compared with vehicle (PBS)-treated controls (*p* < 0.0001 and *p* = 0.0003, respectively; nested one-way ANOVA; Fig. 1a and b) whereas CNPase expression was unchanged (Fig. 1a and b). Cerebellar sections were then stained with MBP to assess the extent of myelination. Compared with wild-type comparators, MBP-positive white matter qualitatively appeared thinner in vehicle-treated *Npc1*^−/−^ mice, a phenotype which improved with the treatment of either rhHSP70 or bimoclomol (Fig. 1c). To quantify the changes in MBP-positive white matter, we measured the area of MBP-positive white matter in a late degenerating cerebellar lobule (lobule VIII) as a proportion of the total lobular area.^43^ Treatment with either rhHSP70 or bimoclomol significantly increased cerebellar white matter thickness compared with vehicle-treated *Npc1*^*−/−*^ mice (*p* < 0.0001 and *p* = 0.0003, respectively; nested one-way ANOVA; Fig. 1d).

### Treatment with rhHSP70 and bimoclomol improves myelination formation in *Npc1*^*−/−*^ mice

**Fig. 2:**
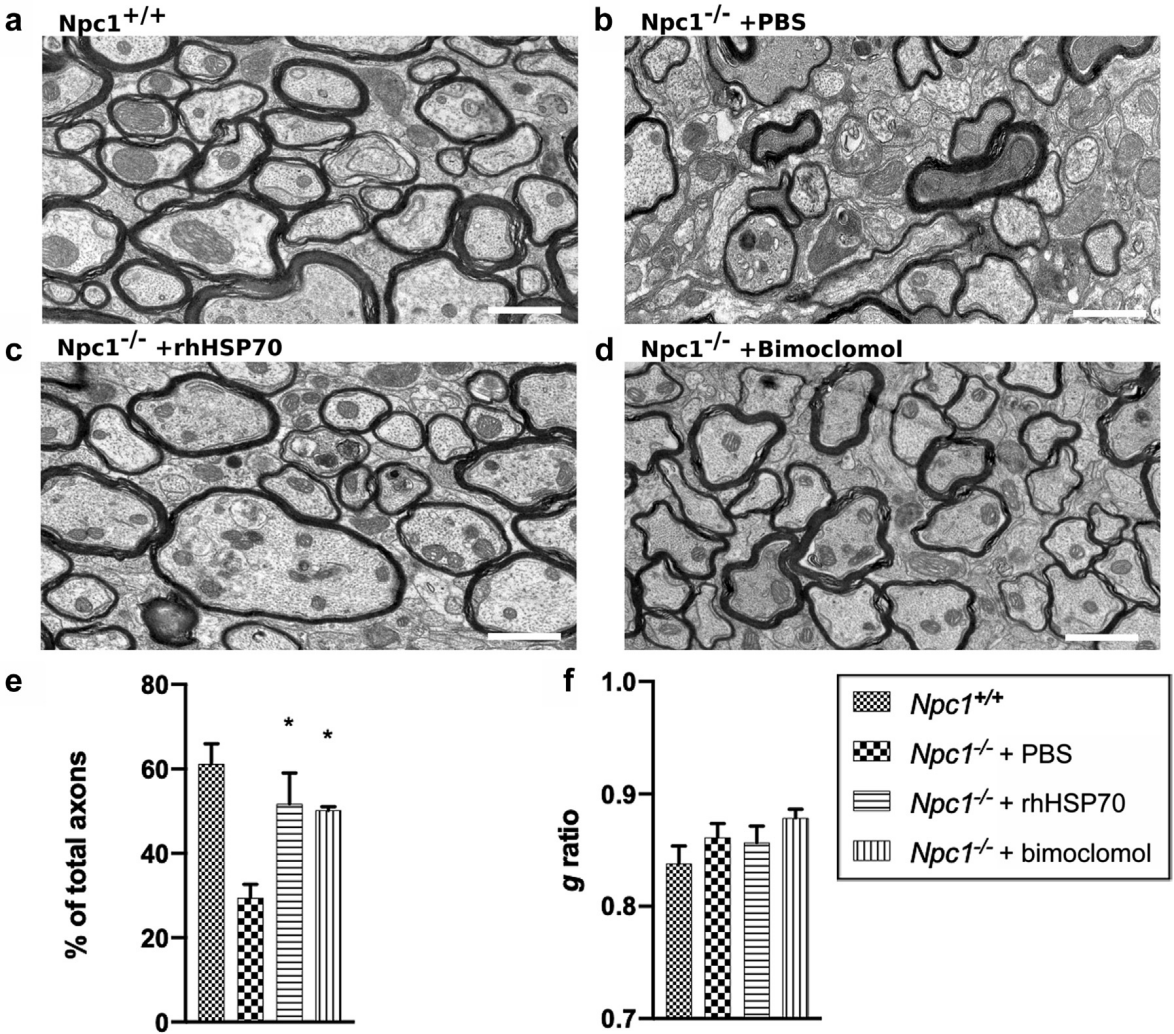
**Treatment with HSP70 and bimoclomol improves myelination formation in *Npc1*^*−/−*^ mice**. Electron micrographs of cerebellar white matter in a) untreated *Npc1*^*+/+*^ mice and *Npc1*^*−/−*^ mice treated with b) PBS, c) rhHSP70, or d) bimoclomol. Scale bar represents 1 μm. e) Myelinated axons as a percentage of total axons in each field of view f) *g*-ratios calculated from >50 randomly selected myelinated axons. Data are shown as mean + SD from 3 to 4 images per animal from a total of n = 2 animals per treatment group. Effects of treatments were analysed using a nested one-way ANOVA on log-transformed values and multiplicity was adjusted using Dunnett's method (n = 2 comparisons; ∗*p* < 0.05, ∗∗*p* < 0.01, ∗∗∗*p* < 0.001, ∗∗∗∗*p* < 0.0001).

To assess whether the increases in MBP levels following treatment with rhHSP70 or bimoclomol improved the formation of functional myelin, we performed TEM on cerebellar lobule VIII sections as this is a lobule that degenerates late in the disease course,^19^ so axons are preserved longer and thus removes degeneration as a confounding factor. Wild-type mice displayed well-myelinated white matter tracts (Fig. 2a), and approximately 60% of the total visible axons were myelinated (Fig. 2e). In contrast, vehicle-treated *Npc1*^*−/−*^ mice exhibited reduced numbers of myelinated axons (Fig. 2b) with only 30% of visible axons myelinated (Fig. 2e). *Npc1*^*−/−*^ mice treated with either rhHSP70 (Fig. 2c) or bimoclomol (Fig. 2d) displayed significantly improved numbers of myelinated axons compared with vehicle-treated controls (*p* = 0.022 and *p* = 0.026, respectively; nested one-way ANOVA; Fig. 2e). Values calculated for the *g*-ratio, a structural and functional index of axonal myelination, showed that of the axons which were myelinated, there were no significant differences between *Npc1*^*−/−*^ mice treated with PBS and either treatment group (Fig. 2f).

### Amplification of heat shock proteins normalises the number of mature OLs in the cerebellum and increases the population of immature OLs in the cortex of *Npc1*^*−/−*^ mice

**Fig. 3:**
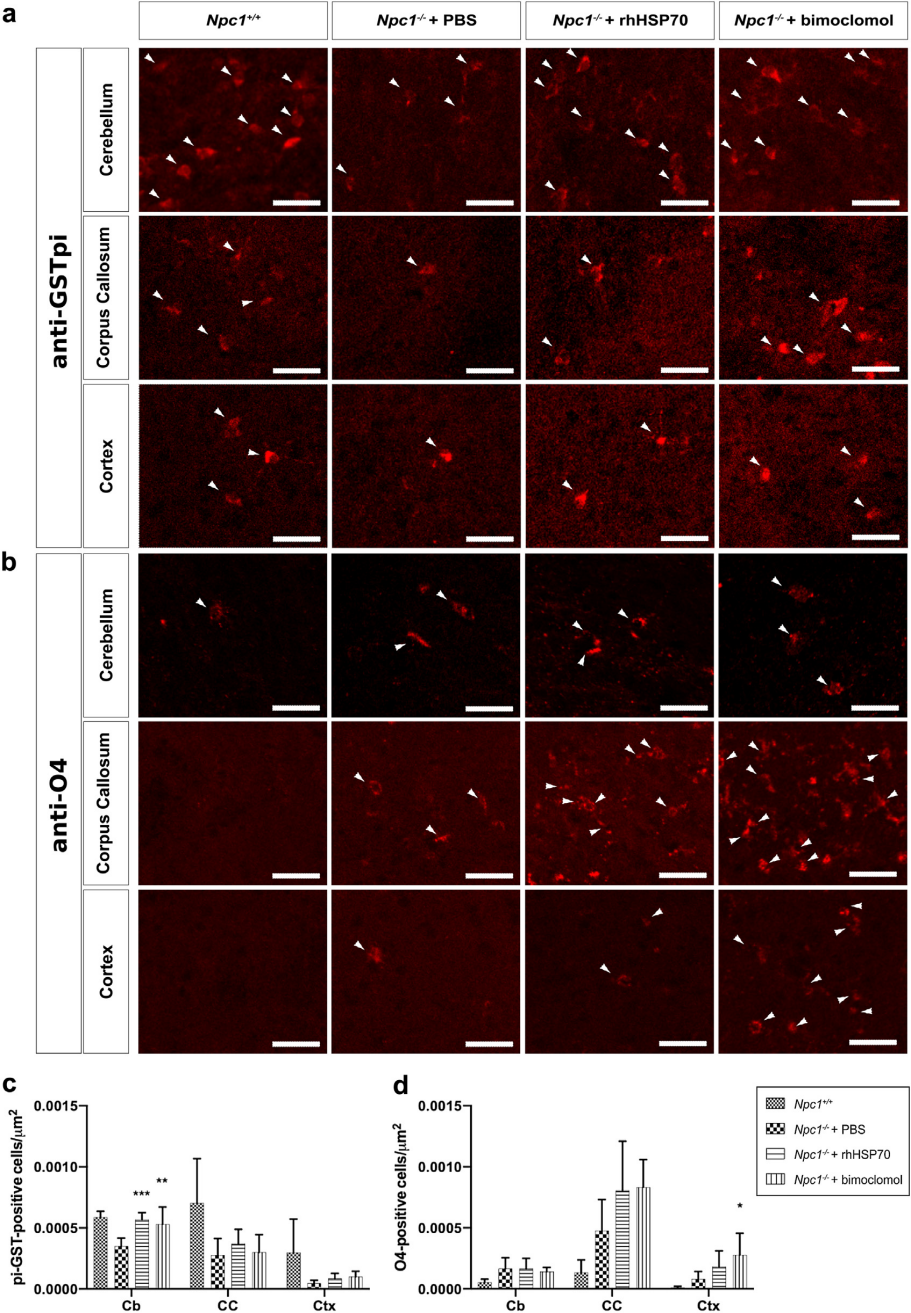
**Amplification of heat shock proteins normalises the number of mature OLs in the cerebellum and increases the population of immature OLs in the forebrain of *Npc1*^*−/−*^ mice**. Representative images of OL lineage cells stained for a) pi-GST and b) O4 in the cerebellum and forebrain of *Npc1*^*+/+*^ mice or *Npc1*^*−/−*^ mice treated with PBS, rhHSP70, or bimoclomol. White arrowheads indicate representative pi-GST or O4-positively labelled OL lineage cells. Scale bars represent 30 μm. Quantification of cells positively labelled for c) pi-GST and d) O4 in the cerebellum (Cb), corpus callosum (CC) and cortex (Ctx). Data are shown as mean + SD of the number of labelled cells per square micron. Effects of treatments were analysed by a nested one-way ANOVA on log-transformed values and multiplicity was adjusted using Dunnett's method (n = 2 comparisons; ∗*p* < 0.05, ∗∗*p* < 0.01, ∗∗∗*p* < 0.001, ∗∗∗∗*p* < 0.0001). *Npc1*^*+/+*^, n = 4; *Npc1*^*−/−*^ + PBS, n = 6; *Npc1*^*−/−*^ + rhHSP70, n = 6; *Npc1*^*−/−*^ + bimoclomol, n = 5. For images stained for pi-GST, 3–7 images per animal were analysed. For images stained for O4 in treated *Npc1*^*−/−*^ mice, 3–5 images per animal were analysed and 1–3 images per animal were analysed from *Npc1*^*+/+*^ mice.

The hypomyelination observed in *Npc1*^*−/−*^ mice has been suggested to be due to impaired differentiation of OL lineage cells.^17^ To assess whether the treatment effects of both rhHSP70 and bimoclomol were due to changes in the number of OLs, we stained for the mature OL marker pi-GST (Fig. 3a) and immature OL marker O4 (Fig. 3b), and co-stained with MBP to delineate total white matter area in the respective CNS regions (Fig. S1a and b). Vehicle-treated *Npc1*^*−/−*^ mice exhibited reductions in the numbers of pi-GST-positive OLs within the cerebellum when compared with wild-type animals (Fig. 3c). Consistent with the improvement of myelination, treatment with either rhHSP70 or bimoclomol normalised the number of pi-GST-positive OLs within the cerebellum (*p* = 0.0009 and *p* = 0.0051, respectively; nested one-way ANOVA; Fig. 3c). The development of OLs from an immature to mature phenotype has been shown to be impaired in *Npc1*^*−/−*^ mice, resulting in an increase in the number of pre-myelinating OLs, particularly within the forebrain.^17^ We observed an increase in the number of O4-positive preoligodendrocyte cells in vehicle-treated *Npc1*^*−/−*^ mice (Fig. 3d). Surprisingly, treatment with bimoclomol significantly increased the numbers of O4-positive OLs within the cortex (*p* = 0.0316: nested one-way ANOVA) of *Npc1*^*−/−*^ mice, suggesting an expansion of the immature OL population (Fig. 3d).

### Heat shock protein amplification increases the ratio of active-to-inactive phosphorylated forms of Fyn kinase in the cerebellum of *Npc1*^*−/−*^ mice

Fyn kinase is a critical regulator of myelination and its activity is primarily controlled through phosphorylation of two tyrosine residues, tyrosine 418 (Y418) and tyrosine 531 (Y531).^24^, ^25^, ^26^

**Fig. 4:**
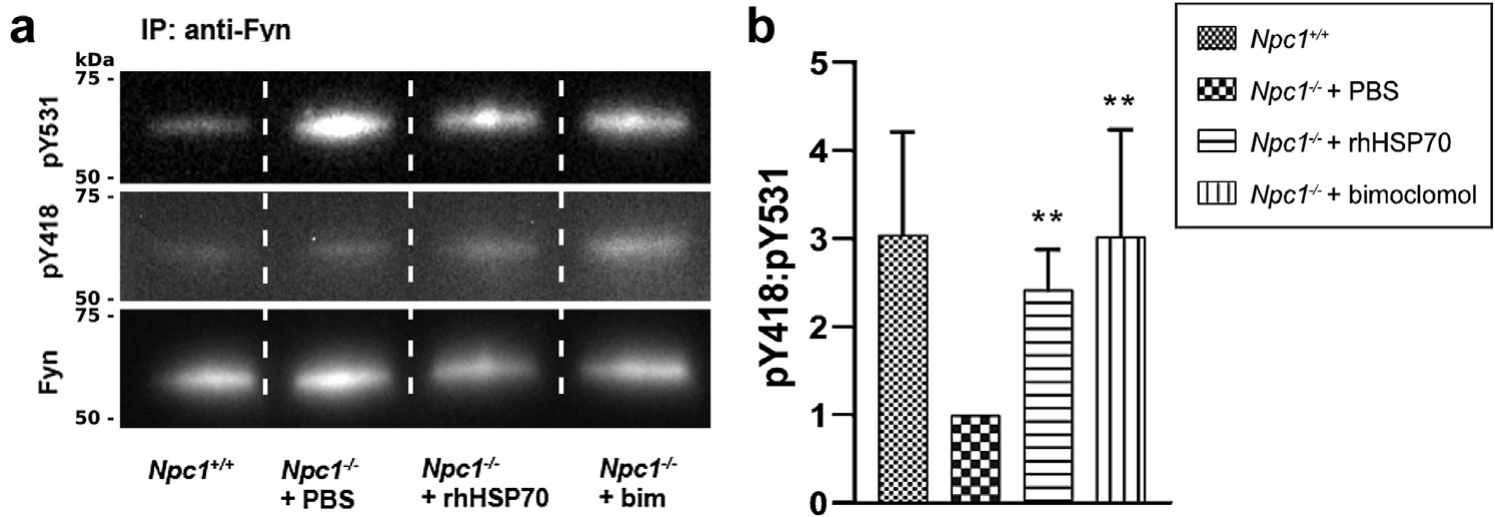
**Treatment with rhHSP70 or bimoclomol increases the ratio of active-to-inactive phosphorylated forms of Fyn kinase in the cerebellum of *Npc1*^*−/−*^ mice**. Representative Western blots of total Fyn and its phosphorylated active (pY418) and inactive (pY531) forms following immunoprecipitation of Fyn kinase in a) cerebellar samples from *Npc1*^*+/+*^ comparators or *Npc1*^*−/−*^ mice following treatment with PBS (vehicle), rhHSP70, or bimoclomol (bim) at P35. b) Quantification of the relative levels of the phosphorylated active and inactive forms of Fyn kinase as well as the ratio of pY418:pY531. Data are presented as the mean + SD of three independent experiments using n = 3 mice per treatment group. Effects of treatments were analysed using a one-way ANOVA on log-transformed values and multiplicity was adjusted using Dunnett's method (n = 2 comparisons; ∗*p* < 0.05, ∗∗*p* < 0.01, ∗∗∗*p* < 0.001, ∗∗∗∗*p* < 0.0001).

To assess whether rhHSP70 and bimoclomol affected the activation of Fyn kinase, we immunoprecipitated Fyn protein in cerebellar samples from wild-type comparators or *Npc1*^*−/−*^ mice treated with PBS (vehicle), rhHSP70, or bimoclomol and probed for the active (pY418) and inactive (pY531) forms of Fyn (Fig. 4a). Vehicle-treated *Npc1*^*−/−*^ mice showed a reduction in the ratio of Fyn pY418:pY531 in the cerebellum (Fig. 4b). Treatment with either rhHSP70 or bimoclomol (*p* = 0.009 and *p* = 0.004, respectively; one-way ANOVA; Fig. 4b) significantly increased this ratio.

### Treatment with bimoclomol improves cerebellar atrophy in *Npc1*^*−/−*^ mice, which is blocked by the Fyn kinase inhibitor saracatinib

**Fig. 5:**
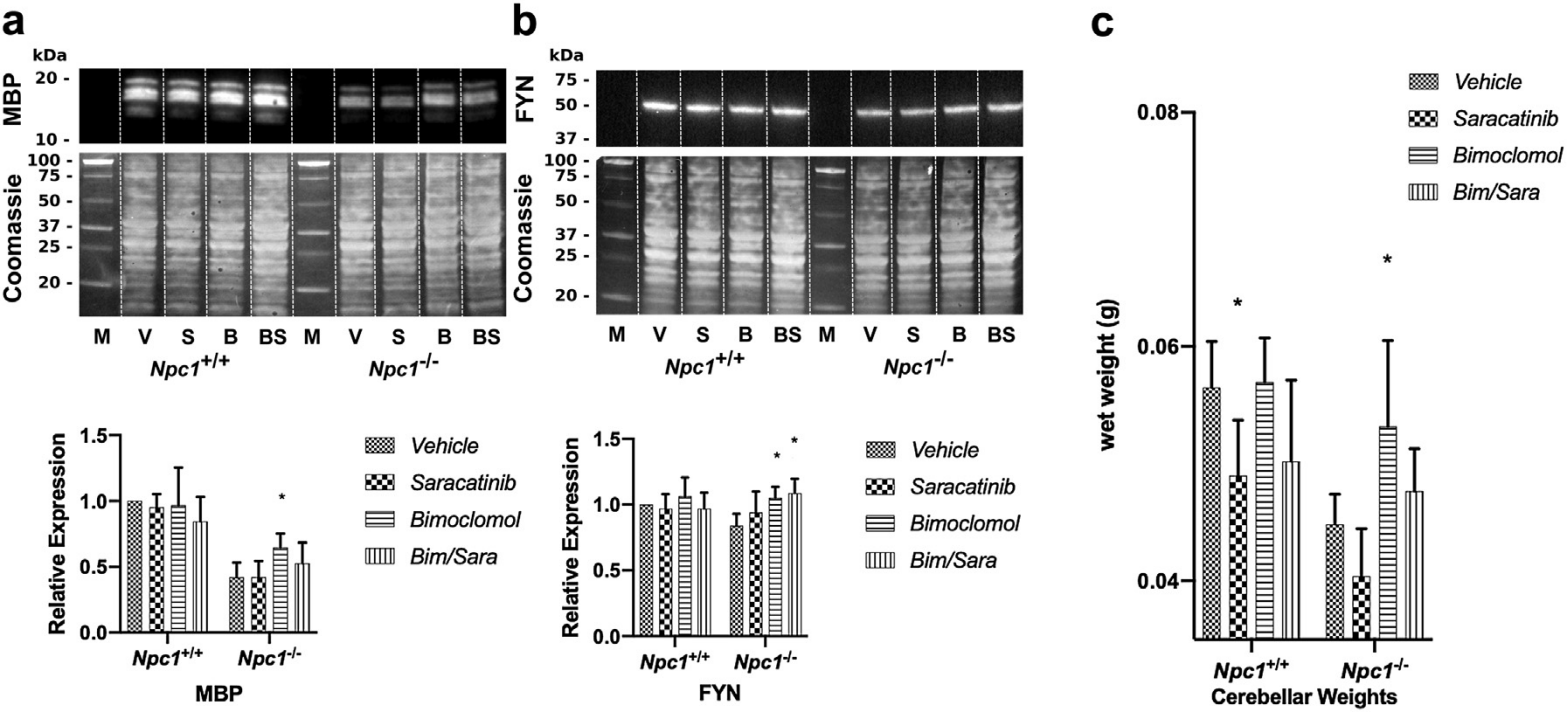
**Bimoclomol mediated improvements in cerebellar weight and myelination are blocked by saracatinib co-treatment**. Representative Western blots & relative expression of a) cerebellar MBP (n = 4/group), b) cerebellar Fyn (n = 4/group) and c) cerebellar wet weights (*Npc1*^*+/+*^ + vehicle: n = 6; *Npc1*^*+/+*^ + saracatinib: n = 6; *Npc1*^*+/+*^ + bimoclomol (Bim): n = 5; *Npc1*^*+/+*^ + bim/sara: n = 5; *Npc1*^*−/−*^ + vehicle: n = 6; *Npc1*^*−/−*^ + saracatinib: n = 5; *Npc1*^*−/−*^ + bimoclomol (Bim): n = 5; *Npc1*^*−/−*^ + bim/sara: n = 6) in *Npc1*^*+/+*^ or *Npc1*^*−/−*^ mice at P35 following treatment with vehicle, 10 mg/kg/day of either saracatinib or bimoclomol, or a combination of bimoclomol/saracatinib (bim/sara). Data are presented as mean + SD. Effects of treatments were analysed using a two-way ANOVA on log-transformed values. Multiplicity was adjusted using Dunnett's method (n = 3 comparisons; ∗*p*< 0.05, ∗∗*p*< 0.01, ∗∗∗*p*< 0.001, ∗∗∗∗*p* < 0.0001).

To determine the contribution of Fyn kinase on the improvement of the myelination defect in *Npc1*^*−/−*^ mice, we administered the Src family kinase inhibitor saracatinib, which has a half maximal inhibitory concentration (IC_50_) of 10 nM for Fyn kinase^49^ and has shown preclinical efficacy in a mouse model of Alzheimer's disease.^55^^,^^56^ Whilst treatment with saracatinib alone did not affect the expression of MBP in *Npc1*^*−/−*^ mice, bimoclomol treatment again significantly increased the expression of MBP in *Npc1*^*−/−*^ mice when compared to vehicle treated *Npc1*^*−/−*^ mice (*p* = 0.028; two-way ANOVA; Fig. 5a). This increase was blocked by coadministration of saracatinib with bimoclomol (Fig. 5a). Interestingly, the significant increase in Fyn expression observed in *Npc1*^*−/−*^ mice treated with bimoclomol was not affected by saracatinib co-administration (Fig. 5b). Treatment with either saracatinib or bimoclomol alone or in combination did not alter the expression of MBP or Fyn in *Npc1*^*+/+*^ mice. Vehicle-treated *Npc1*^*−/−*^ mice exhibited a reduction in cerebellar weights compared with those of vehicle-treated wild-type mice (Fig. 5c). Furthermore, treatment with saracatinib resulted in a significant reduction of cerebellar weights in wild-type mice compared with those of vehicle-treated wild-type mice (*p* = 0.041; two-way ANOVA; Fig. 5c). Bimoclomol administered both alone and in combination with saracatinib did not have a significant effect on cerebellar weights compared to control in wild-type mice. Interestingly, in *Npc1*^*−/−*^ mice, treatment with bimoclomol prevented cerebellar atrophy and preserved cerebellar weights at wild-type weights (*p* = 0.025; two-way ANOVA), whereas this improvement was blocked by co-administration with saracatinib (Fig. 5c). Treatment with saracatinib alone resulted in a small, non-significant decrease in cerebellar weights.

## Discussion

Our findings suggest that amplification of HSPs may be a promising strategy for treating hypomyelination in NPC, a progressive and prematurely fatal neurovisceral disease with limited therapeutic options. We have utilised the *Npc1*^nih^ mouse model to address whether direct application of rhHSP70 or drug-modulated HSP amplification with the arimoclomol-analogue bimoclomol could improve the prominent hypomyelination observed within the CNS. The current study was motivated by previous findings showing that treatment with rhHSP70 in *Npc1*^*−/−*^ mice significantly improved cerebellar white matter thickness and MBP levels as well as the behavioural phenotypes associated with NPC.^43^

Here, we demonstrate that treatment with either rhHSP70 or bimoclomol improves cerebellar myelination in *Npc1*^*−/−*^ mice, as evidenced by increased MBP levels and myelin formation. CNPase, another component of myelin was unchanged by treatment, the reason for this is unclear. CNPase is both present in premyelinating OLs as well as activated microglia.^57^ Reductions observed in NPC disease therefore may not be due only to differences in OL CNPase expression but deficiencies in other cell types as well. Interestingly, CNPase deficient mouse models show no overt myelin defects.^57^ Both treatment with rhHSP70 and bimoclomol normalised the numbers of mature OLs and increased the ratio of active-to-inactive forms of phosphorylated Fyn kinase in the cerebellum. Furthermore, treatment with bimoclomol improved MBP expression, prevented cerebellar atrophy and preserved cerebellar weights at wild-type weights in a separate cohort of *Npc1*^*−/−*^ mice; these effects were blocked by co-administration with the Fyn kinase inhibitor, saracatinib. The improvements were associated with a small but significant increase in Fyn expression in *Npc1*^*−/−*^ mice treated with bimoclomol. Finally, bimoclomol-treated mice exhibited an expansion of the immature OL population in the cortex, an effect which seems insufficient on its own to initiate significant myelination at this stage of development. Taken together, these findings suggest that HSP amplification in *Npc1*^*−/−*^ mice improves cerebellar myelination and rescues cerebellar atrophy by normalising the population of mature OLs required for proper myelin formation, potentially through a Fyn kinase-mediated mechanism though further work is needed to confirm that saracatinib blocked myelination through inhibition of Fyn phosphorylation.

The association between impaired OL maturation and hypomyelination in disease models of NPC has been reported by others^15^, ^16^, ^17^^,^^32^^,^^58^^,^^59^; however, the underlying mechanisms are not fully understood. Several explanations have been posited including the downregulation of transcriptional regulators governing OL differentiation^15^^,^^16^ and impaired signalling pathways, including through polysialylated axonal NCAM,^16^ altered adenosine A_2A_ receptor function^57^ and aberrant phosphorylation of Fyn kinase.^17^^,^^32^^,^^59^ In the current study, treatment with either rhHSP70 or bimoclomol improved myelination and normalised the numbers of mature OLs within the cerebellum of *Npc1*^*−/−*^ mice, a finding that was associated with increased activation of Fyn, whose activity is mediated in part by the interaction between the α6β1-integrin complex and laminin-2 on the cell membrane of proliferating OLs.^24^ Interestingly, reduced cell surface expression of β1-integrins has been observed in NPC1 mutant fibroblasts, likely due to alterations in intracellular trafficking and subsequent recycling to the plasma membrane as a result of the low density lipoprotein-derived cholesterol sequestered within late endosomes.^60^^,^^61^ Given the critical role of integrins in cell migration and adhesion,^62^ it is possible that a reduction in β1-integrins on the cell surface of proliferating OLs could explain the observed impairments in OL maturation in *Npc1*^*−/−*^ mice. The mature OLs are likely responsible for the functional improvements in cerebellar myelination; however, it is unclear whether the mature OL pool is driven by the increased number of forebrain-derived immature OLs, a population that could potentially migrate to the cerebellum during development^63^ or whether this expansion is the result of increased maturation signalling to the OLs. The reason why the large population of immature OLs in the forebrain do not translate into mature OLs is not clear. A previous study of HSP70 in *Npc1*^*−/−*^ mice also indicated that forebrain myelin was not improved.^43^ Whether different signals for myelination exist between forebrain and cerebellum, or this is due to differences in disease progression between the two regions in NPC disease remains to be determined. A further point to note is that myelin is a lipid dense structure and the effects observed in this study may be due to alterations in lipid homeostasis, a previously observed effect of HSP70 in NPC. A limitation of this study is that we are unable to confirm the impact of hypomyelination on disease progression relative to other contributing pathologies. Further studies could be undertaken to determine the impact of myelination on disease course, and to elucidate the relative contributions of lipid metabolism, OL numbers and Fyn phosphorylation to the observed effects.

The above findings strongly suggest that amplification of HSPs may hold promise for treating the myelination impairments in NPC, a pathological process that has been associated with ataxia and saccadic gain in adult NPC patients.^9^ One question that remains to be answered is the role of demyelination in the neuropathology of NPC. Rescue of myelination clearly improves behavioural outcomes and prevents the cerebellum from atrophy, but it is not known whether the improvements in functional myelin can prevent the degeneration of cerebellar Purkinje neurons, a pathological process that has been observed in both murine and feline models of NPC.^18^^,^^19^^,^^64^^,^^65^ Widespread reductions in white matter tracts at the expense of localised reductions in grey matter in adult NPC patients^8^ and neuronal loss in a patient with late infantile onset NPC^7^ support the hypothesis that impairments in myelination and axonal morphology may precede the neurodegeneration in NPC.

The current therapeutic options for treating NPC are limited. Miglustat has been shown to slow the loss of white matter in adolescent and adult-onset NPC patients who received treatment for a median of 2.8 years.^38^^,^^39^ Here, we show that treatment with both rhHSP70 and the arimoclomol-analogue bimoclomol improves functional myelin formation *in vivo*. A recent clinical phase II/III trial with arimoclomol in NPC showed significant increases in the levels of HSP70, reduction of biomarkers of disease burden and clear reduction in clinical disease progression suggesting that amplification of HSPs may be a promising strategy for treating NPC.^44^

In summary, we suggest that HSP-amplifying compounds constitute a viable first-in-class therapeutic option for NPC, a progressive disease for which there is a shortage of effective treatments.

## Contributors

Study design: J.G., T.K., F.M.P. Data collection: J.G., M.E.F.-S., M.F., D.S., C.S., E.K. Data analysis: J.G., M.E.F.-S., M.F., E.K., A.M.P., C.K.F. Data interpretation: J.G., A.M.P., C.K.F., T.K., F.M.P. Data validation: J.G., M.E.F.-S., A.M.P. Figures: J.G., M.F., A.M.P., F.M.P. Writing: J.G., A.M.P., C.K.F., T.K., F.M.P. All authors read and approved the final version of the manuscript.

## Data sharing statement

All data needed to understand the methodology and evaluate the conclusions in the paper are present in the paper.

## Declarations of interests

A.M.P., C.K.F., and T.K. are former employees of Orphazyme A/S. T.K. holds shares in Orphazyme A/S. T.K. is a founder of Orphazyme A/S. J.G. was funded in part by Orphazyme A/S. M.F. and M.E.F.-S. were funded by Orphazyme A/S. F.M.P. is a former consultant to Orphazyme A/S. C.S., D.S. and E.K. declare no conflict of interests.

## Appendix A Supplementary data

Fig. S1 Amplification of heat shock proteins normalises the number of mature OLs in the cerebellum and increases the population of immature OLs in the forebrain of *Npc1*^*−/−*^ mice. Representative images of OL lineage cells stained for a) pi-GST & MBP and b) O4 & MBP in the cerebellum and forebrain of *Npc1*^*+/+*^ mice or *Npc1*^*−/−*^ mice treated with PBS, rhHSP70, or bimoclomol. White arrowheads indicate representative pi-GST- or O4-positively labelled OL lineage cells. Scale bars represent 30 μm. *Npc1*^*+/+*^, n = 4; *Npc1*^*−/−*^ + PBS, n = 6; *Npc1*^*−/−*^ + rhHSP70, n = 6; *Npc1*^*−/−*^ + bimoclomol (Bim), n = 5. For images stained for pi-GST, 3–7 images per animal were analysed. For images stained for O4 in treated *Npc1*^*−/−*^ mice, 3–5 images per animal were analysed and 1–3 images per animal were analysed from *Npc1*^*+/+*^ mice.
