# Supplementary figures and images for: Heat shock protein amplification improves cerebellar myelination in the Npc1nih mouse model
Source: eBioMedicine. 2022 Nov 28;86:104374. doi: 10.1016/j.ebiom.2022.104374 (PMC9713282; doi:10.1016/j.ebiom.2022.104374)

a

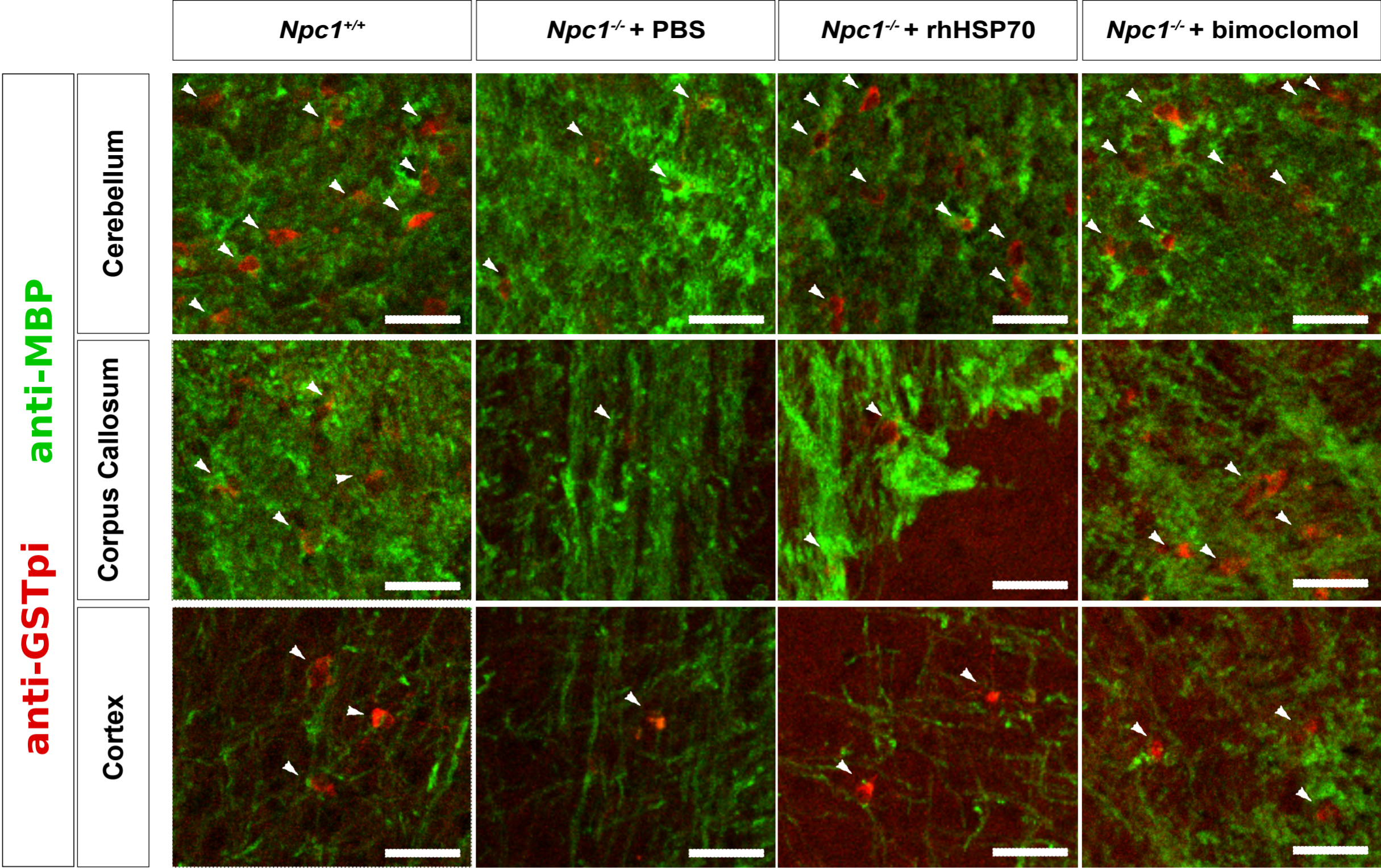

b

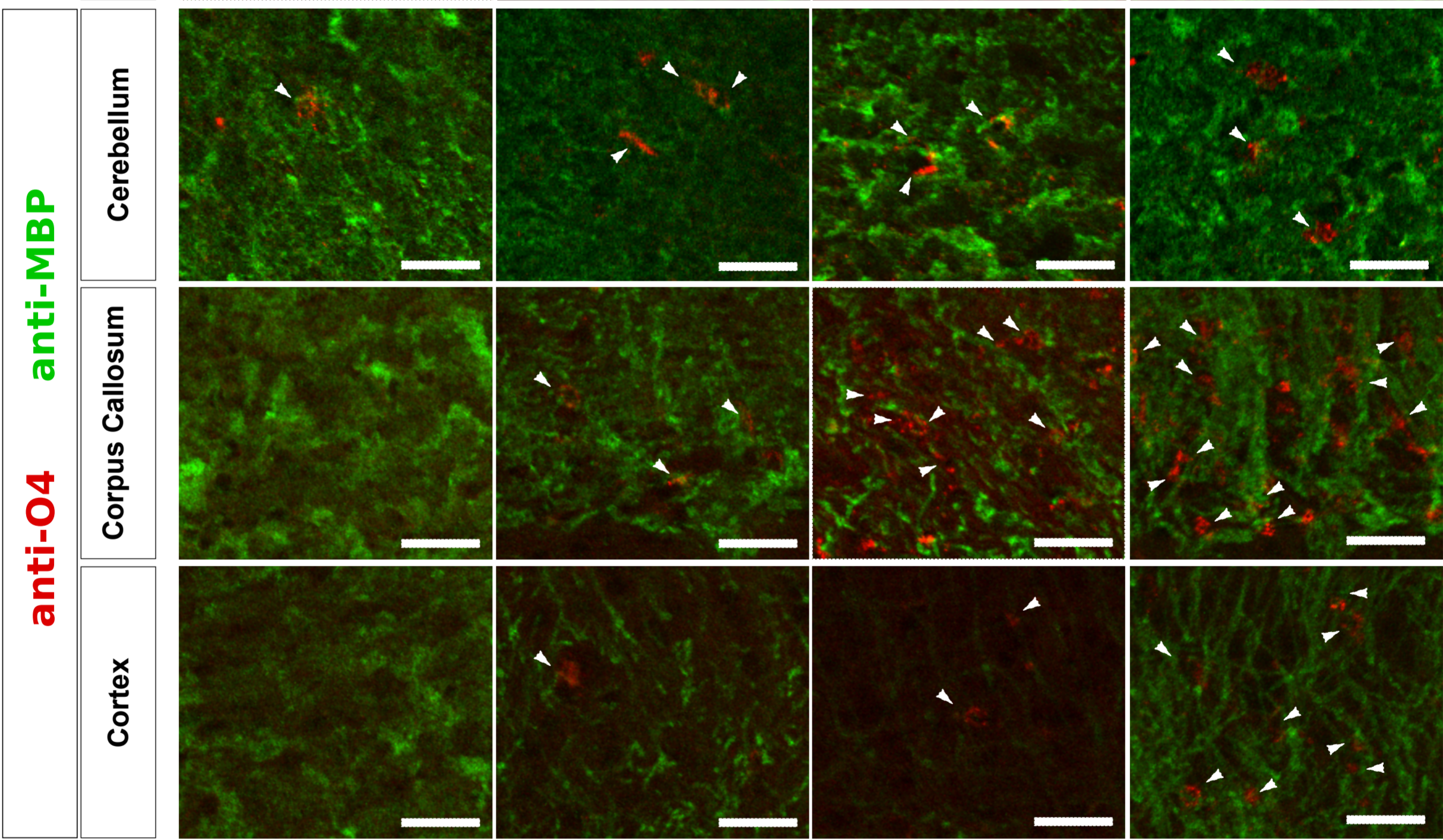

Supplement: Fig. S1 — Amplification of heat shock proteins normalises the number of mature OLs in the cerebellum and increases the population of immature OLs in the forebrain of Npc1−/− mice. Representative images of OL lineage cells stained for a) pi-GST & MBP and b) O4 & MBP in the cerebellum and forebrain of Npc1+/+ mice or Npc1−/− mice treated with PBS, rhHSP70, or bimoclomol. White arrowheads indicate representative pi-GST- or O4-positively labelled OL lineage cells. Scale bars represent 30 μm. Npc1+/+, n = 4; Npc1−/− + PBS, n = 6; Npc1−/− + rhHSP70, n = 6; Npc1−/− + bimoclomol (Bim), n = 5. For images stained for pi-GST, 3–7 images per animal were analysed. For images stained for O4 in treated Npc1−/− mice, 3–5 images per animal were analysed and 1–3 images per animal were analysed from Npc1+/+ mice. [file mmc1.pdf]
